# Supplementary figures and images for: Efferocytosis is restricted by axon guidance molecule EphA4 via ERK/Stat6/MERTK signaling following brain injury
Source: J Neuroinflammation. 2023 Nov 9;20:256. doi: 10.1186/s12974-023-02940-5 (PMC10633953; doi:10.1186/s12974-023-02940-5)

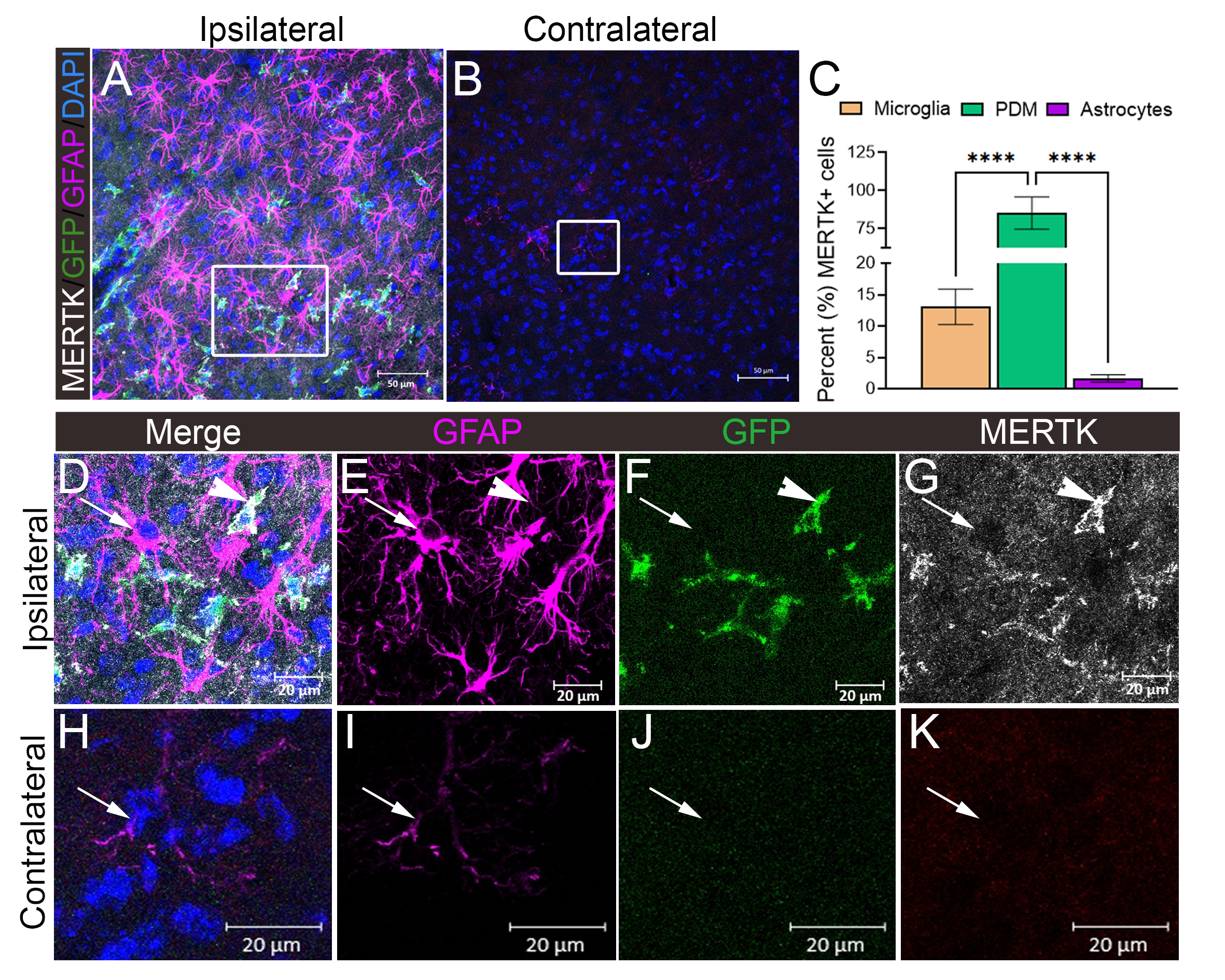

Supplement: Supplementary file 1 — Additional file 1: Figure S1. Low MERTK Expression in astrocytes within the ipsilateral cortex of CCI-injured GFP+ bone marrow chimeric wild type (WT+WTBMCs) mice. MERTK (white) is upregulated in GFP+ (green, arrowhead) peripheral-derived immune cells and not in the GFAP + (purple) astrocytes in the ipsilateral cortex at 3dpi (A& D-G). The contralateral cortex shows minimal GFAP (purple) expression in astrocytes (arrow) and no detectable MERTK + or GFP + cells (B& H–K). C) The percentage of MERTK-expressing GFP-IBA1 + microglia, GFP + IBA1 + PDMs, and GFAP + astrocytes was quantified in the ipsilateral cortex at 3dpi using the optical fractionator probe function of Stereoinvestigator. N = 3–5 mice/group. ****P < 0.0001. One-way ANOVA followed by Tukey’s multiple comparisons test. Scale bar = 50 µm in A, B and 20 µm D-K. [file 12974_2023_2940_MOESM1_ESM.tif]

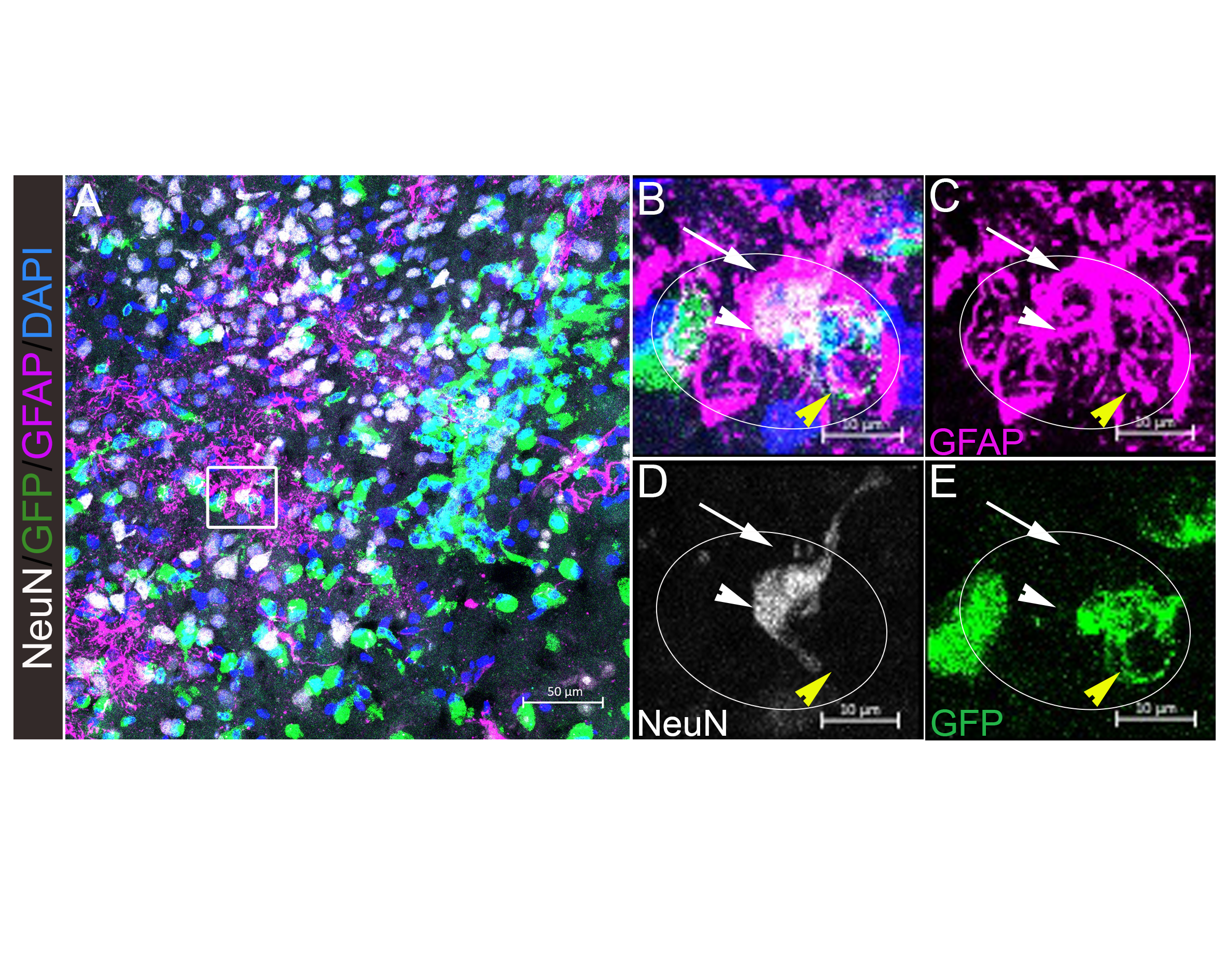

Supplement: Supplementary file 2 — Additional file 2: Figure S2. Astrocytes approach and wrap neurons and peripheral immune cells within the ipsilateral cortex of CCI-injured GFP + bone marrow chimeric wild-type (WT+WTBMCs) mice. (A) Representative confocal images for GFAP (purple)- and NeuN (white)-stained coronal section of WT+WTBMCs mice. (B-E) Inset from A, showing GFAP + astrocyte’s processes are wrapping GFP + peripheral-derived immune cells (yellow arrowhead) and NeuN + (white) neurons (white arrowhead) in the peri-lesion cortex at 3dpi. Scale bar = 50 µm in A and 20 µm in B-E. [file 12974_2023_2940_MOESM2_ESM.tif]

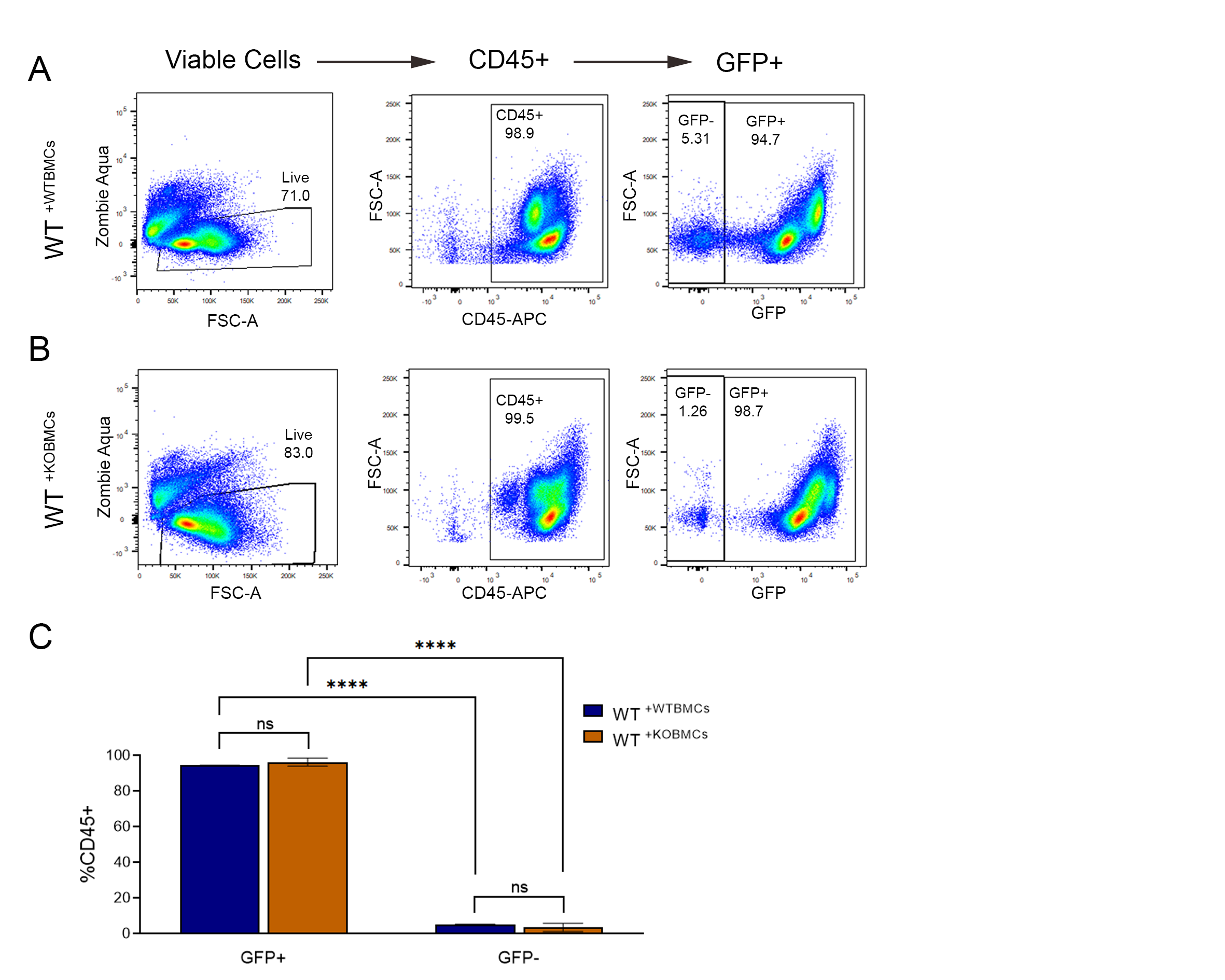

Supplement: Supplementary file 3 — Additional file 3: Figure S3. Chimerism levels in WT+WTBMCs and WT+KOBMCs mice at 1-month after bone marrow transplantation. A-B) Flow cytometry gating strategy to select viable, CD45 +, GFP + , and GFP- leucocytes in the blood of WT+WTBMCs (A) and WT+KOBMCs (B) mice at 1 month following bone marrow transplant. C) Percentage of GFP + and GFP- Cd45 + leucocytes in WT+WTBMCs and WT+KOBMCs mice. [file 12974_2023_2940_MOESM3_ESM.tif]

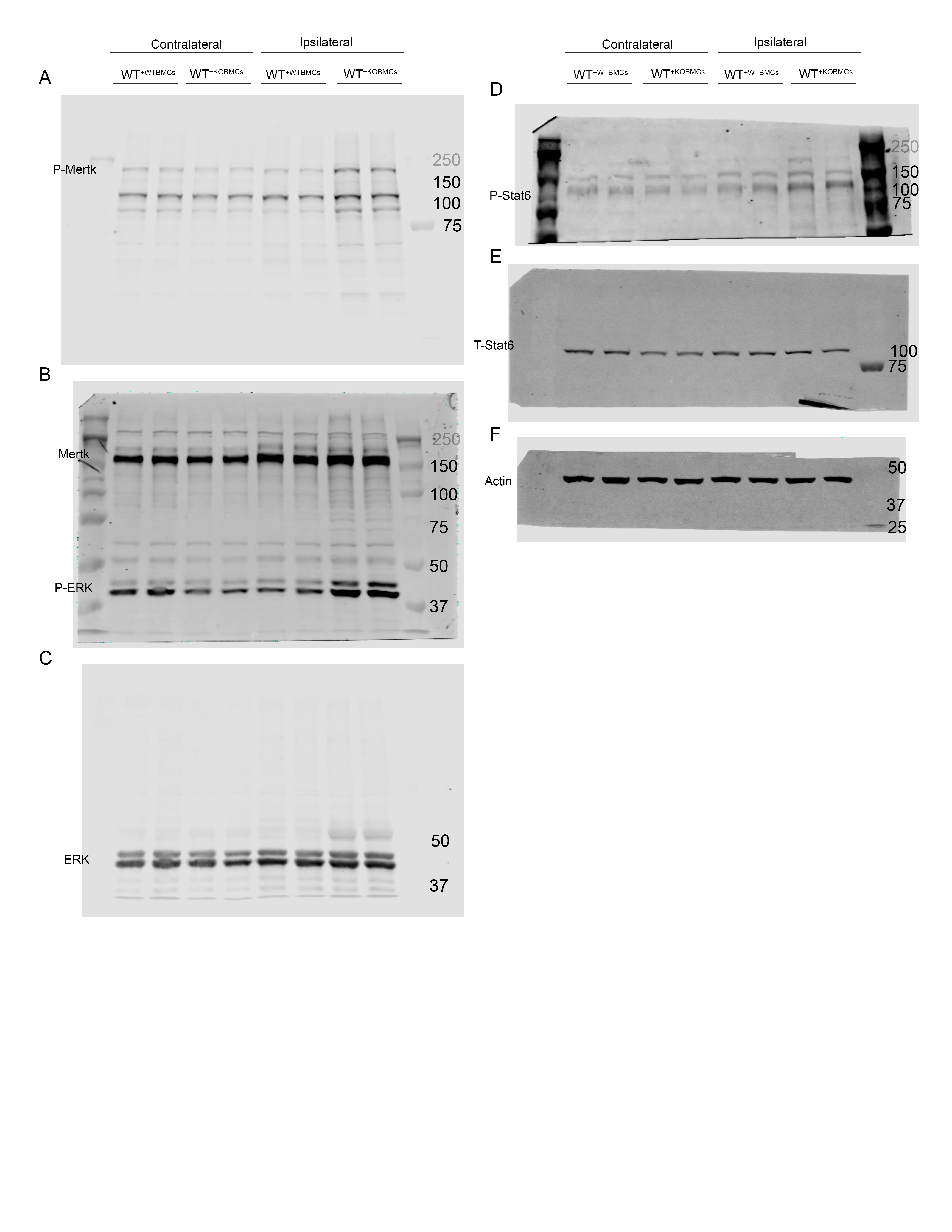

Supplement: Supplementary file 4 — Additional file 4: Figure S4. Western blot analysis shows the expression of P-MERTK (A), MERTK (B), P-ERK1/2 (B), ERK1/2 (C), and P-Stat6 (D), Stat6 (E), and Actin (F) in the contralateral and ipsilateral cortex of chimeric WT+WT BMCs and WT+KO BMCs mice at 3dpi. [file 12974_2023_2940_MOESM4_ESM.tif]

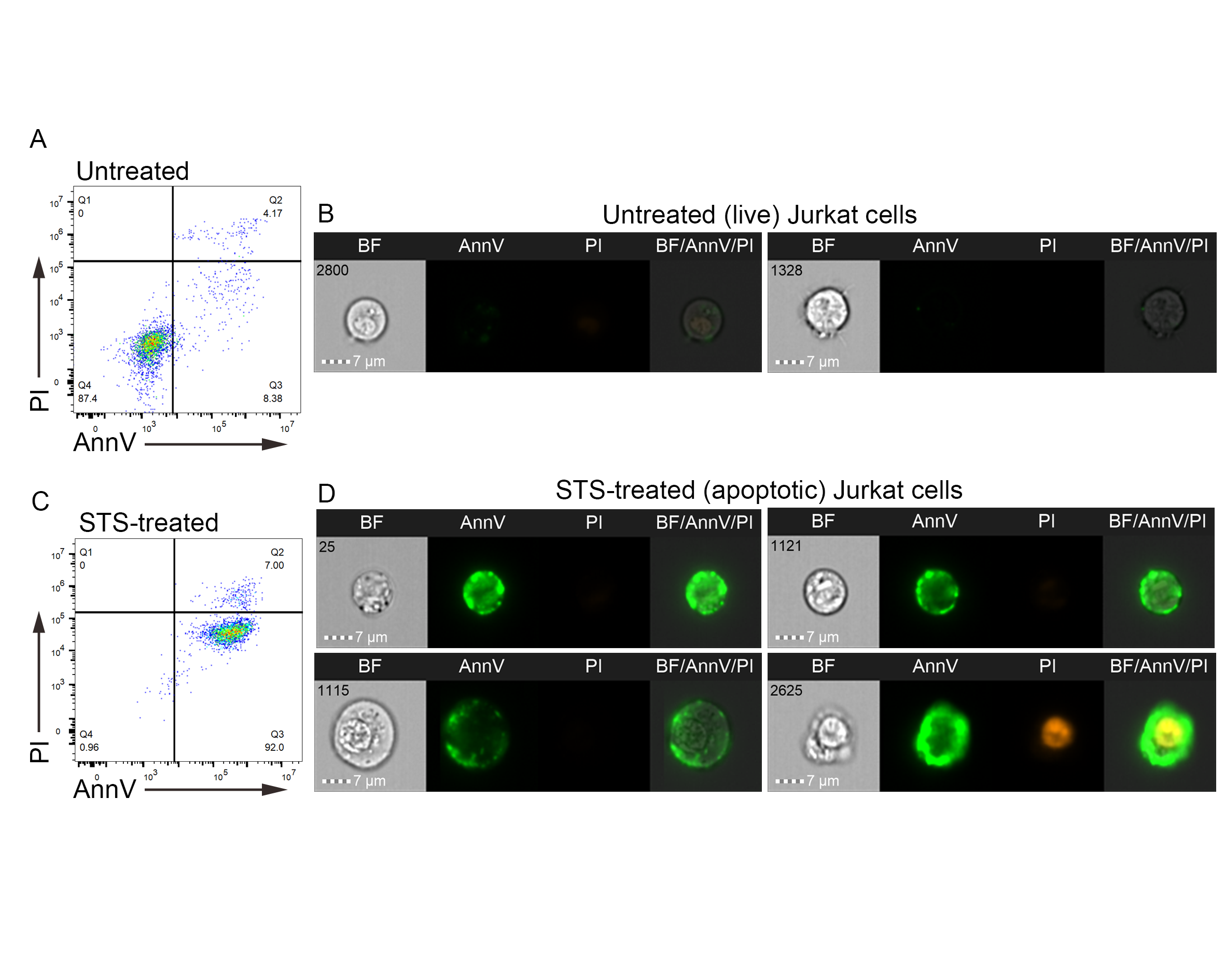

Supplement: Supplementary file 5 — Additional file 5: Figure S5. Induction of apoptosis in Jurkat cells by staurosporine for in vitro efferocytosis experiment. Jurkat cells were treated with 1 μM of Staurosporine (STS) for 3 h and stained with Annexin V (AnnV) and Propidium Iodide (PI). A, C) Representative flow cytometry plots showing the percentage of live (AnnV− PI−, Q4), early apoptotic (AnnV+ PI−, Q3), and late apoptotic (AnnV+PI+, Q2) Jurkat cells in the absence (A) or presence (C) of STS treatment. B, D) Representative images taken by ImageStream flow cytometry for live (B) and apoptotic (D) Jurkat cells stained with AnnV (green) and PI (red). [file 12974_2023_2940_MOESM5_ESM.tif]
